# Parkinson’s Disease: Available Clinical and Promising Omics Tests for Diagnostics, Disease Risk Assessment, and Pharmacotherapy Personalization

**DOI:** 10.3390/diagnostics10050339

**Published:** 2020-05-25

**Authors:** Oxana P. Trifonova, Dmitri L. Maslov, Elena E. Balashova, Guzel R. Urazgildeeva, Denis A. Abaimov, Ekaterina Yu. Fedotova, Vsevolod V. Poleschuk, Sergey N. Illarioshkin, Petr G. Lokhov

**Affiliations:** 1 Laboratory of mass spectrometry-based metabolomics diagnostics, Institute of Biomedical Chemistry, 10 building 8, Pogodinskaya street, 119121 Moscow, Russia; dlmaslov@mail.ru (D.L.M.); balashlen@mail.ru (E.E.B.); lokhovpg@rambler.ru (P.G.L.); 2 5th Neurological Department (Department of Neurogenetics), Research Centre of Neurology, Volokolamskoe shosse, 80, 125367 Moscow, Russia; guliaurasgildeeva@gmail.com (G.R.U.); abaidenis@yandex.ru (D.A.A.); ekfed@mail.ru (E.Y.F.); pol82@yandex.ru (V.V.P.); snillario@gmail.com (S.N.I.)

**Keywords:** Parkinson’s disease, diagnostics, clinical test, metabolomics, biomarker, pharmacotherapy personalization

## Abstract

Parkinson’s disease is the second most frequent neurodegenerative disease, representing a significant medical and socio-economic problem. Modern medicine still has no answer to the question of why Parkinson’s disease develops and whether it is possible to develop an effective system of prevention. Therefore, active work is currently underway to find ways to assess the risks of the disease, as well as a means to extend the life of patients and improve its quality. Modern studies aim to create a method of assessing the risk of occurrence of Parkinson’s disease (PD), to search for the specific ways of correction of biochemical disorders occurring in the prodromal stage of Parkinson’s disease, and to personalize approaches to antiparkinsonian pharmacotherapy. In this review, we summarized all available clinically approved tests and techniques for PD diagnostics. Then, we reviewed major improvements and recent advancements in genomics, transcriptomics, and proteomics studies and application of metabolomics in PD research, and discussed the major metabolomics findings for diagnostics and therapy of the disease.

## 1. Introduction

Parkinson’s disease (PD) is one of the most significant problems in clinical neurology, both due to the high prevalence in population around the world and significant the disability of patients [1,2,3]. In accordance with the existing classification, among the parkinsonian syndromes there are distinguished: (1) primary parkinsonism, (2) atypical parkinsonism, (3) secondary parkinsonism, and 4) parkinsonism in hereditary diseases of the central nervous system [4,5]. Primary parkinsonism includes Parkinson’s disease (PD), the second most common neurodegenerative disease that presents a significant medical and socio-economic problem. Currently, the PD’s diagnostics are based on developed clinical criteria, the correct use of which is largely determined by the qualification of the specialist, and therefore the differentiation of the disease from other pathologies may be challenging at early stages [6,7,8]. Identification of markers of the pathological process in PD, the character of its course and prognosis, and the risk of disease developing is considered as a very important task. The most popular diagnostic markers today are substantia nigra hyperechogenicity in transcranial sonography [9,10], hyposmia determined by special quantitative methods [11,12], and objective assessment of color perception, retinal thickness, and oculomotor parameters [13,14,15]. Almost all PD’s biomarkers are considered of use for the diagnostics of early and premotor stages of the disease. However, it should be noted that none of these indicators itself provides comprehensive diagnostic information, and they do not allow the prognosis of the disease to be fully assessed or the diagnosis verified [16].

It is believed that the neurodegenerative processes in PD begin several years or even decades before the onset of motor symptoms that underline the diagnosis [2,17]. In this regard, the development of approaches for early diagnostics of the prodromal phase of the disease, as the most promising from the perspectives of the possibilities to implement neuroprotective strategies and preventive therapy in PD patients, has been of considerable interest in recent years [18]. In 2015, the International Parkinson and Movement Disorder Society (MDS) for the first time summarized research criteria for prodromal Parkinson’s disease, to estimate the probability of prodromal PD [19]. Diagnostics of prodromal PD is based on the presence or absence of risk factors and prodromal markers of the disease. Known risk factors include, for example, gender (the risk of PD developing is higher in men), smoking and caffeine consumption (reduce the risk of disease developing), the presence of a burdened family disease, the presence of mutations and substantia nigra hyperechogenicity, as well as prodromal markers including hyposmia, rapid eye movement (REM) sleep behavior disorder, depression, and autonomic dysfunction [8,19,20].

The study of prodromal markers also allows studying the pathogenesis of the disease, its stages, and neuroplasticity mechanisms at the early stage of pathology developing. Several studies have been initiated in the world to find the optimal combination of prodromal stage biomarkers—both on samples of the general population (a large number of subjects and long follow-up period are required), and on samples of “enriched” subjects consisting of individuals with an already identified risk factor/prodromal marker of the disease [21]. Algorithms being created and improved for prodromal PD diagnostics (as well as other neurodegenerative diseases) are recognized today as one of the most urgent challenges facing neurology.

In this review, we summarized all available clinically approved tests and techniques for PD diagnostics. Then, we reviewed major improvements and recent advancements in genomics, transcriptomics, and proteomics studies and application of metabolomics in PD research, and further discussed the major metabolomics findings for diagnostics and therapy of the disease.

## 2. Available Clinical Tests and Investigations for PD Diagnostics

New diagnostic criteria for PD were developed by the Council of Experts of the International Movement Disorder Society for the study of movement disorders [6]. According to a MDS presented document, the diagnostic criteria for PD are divided into four groups: main criteria for parkinsonism diagnostics as the core feature of the disease, supportive criteria (positive features that increase the confidence of the PD diagnosis), absolute exclusion criteria (which rule out PD), and red flags (which must be counterbalanced by additional supportive criteria to allow diagnosis of PD) (Table 1). Based on the MDS criteria two levels of PD diagnosis are delineated: clinically established PD, where patient displays parkinsonism plus at least two supportive criteria and absence of any exclusion criteria; and probable PD, where in addition to parkinsonism, the absence of exclusion criteria and presence of balanced numbers of supportive criteria and red flags are revealed [6]. The Movement Disorder Society PD criteria are intended for use in clinical research but also may be used to guide clinical diagnosis. The studies have demonstrated high sensitivity and specificity of the MDS criteria in comparison to other standard expert diagnostics, for example, the United Kingdom Brain Bank criteria [7]. For PD diagnosis according to the MDS criteria, a detailed medical history is collected for each patient, including a standard questionnaire and a neurological examination to determine the age of onset, duration of the disease, form, and stage using the Hoehn and Yahr scale. To recognize some criteria the existing pharmacological and instrumental tests are made (Appendix A, Table A1).

### 2.1. Pharmacological Test

The diagnosis of PD is confirmed by a significant persistent effect of dopaminergic therapy. An absence of adequate response to treatment in a dose of 1000 to 1500 mg per day over at least two months strongly suggests that the initial diagnosis of PD was incorrect and should be re-evaluated for one of the other parkinsonian syndromes. The acute levodopa challenge test involves rating motor symptoms of parkinsonism according to the Disorder Society-unified Parkinson’s disease rating scale (MDS-UPDRS) both before and after “single-short” administration of an above-threshold dose of levodopa (for example, carbidopa–levodopa 25/250 mg) or subcutaneous administration of apomorphine (in a dose from 1.5 to 4.5 mg). Although this test is not standardized, it is considered positive in a case of a clinically significant improvement in MDS-UPDRS motor symptoms (usually in the range of 15 to 30 percent or more) in an hour after levodopa administration or 20 min after apomorphine administration [22,23,24].

### 2.2. Instrumental Tests

One of the broadly applied tools for noninvasive diagnostic imaging techniques used in PD diagnostics is transcranial sonography (TCS). The use of TCS in PD is based on an investigation of the morphology of substantia nigra (SN) and assessing substantia nigra hyperechogenicity (SNH) due to its increased iron content [25,26]. In numerous independent studies including blinded experiments, it has been shown that SNH is detected in 84–95% of patients with PD on average [27]. However, the SNH can also be detected in 9% of clinically healthy people (from 8% to 15%) [26,28,29]. According to studies the sensitivity and specificity of the method in differentiating individuals with PD from control patients is 80–93%, and 76–92%, respectively [29,30,31,32]. It is believed that the area of SN does not change and remains stable throughout the disease, despite the clinical deterioration and progression of the process from the initial stages to severe disability [33,34]. This biomarker can be used both for the diagnosis of PD at any stage and as a basis for forming a risk group and establishing a predisposition to the development of PD in individuals before the onset of motor symptoms of the disease [35], and for differential diagnosis with essential tremor (ET) and atypical parkinsonism [9,26,28,36].

The undoubted advantages of the method are low cost, noninvasiveness, short investigation duration, and the possibility to repeat the investigation in dynamics. The TCS limitations include: the dependency of the method on the quality of the temporal acoustic bone window, the requirement of a high-quality ultrasound system, and the investigation should be conducted by a highly qualified specialist with extensive experience on specific TCS equipment (subjectivism in performing and interpreting the results of ultrasound scanning). Furthermore, as the SNH is not a marker of the severity and progression of PD, it cannot be used to assess the degree of neurodegeneration [26].

Functional neuroimaging methods are the only additional research methods that can confirm the presence of PD in a lifetime, detect changes long before the appearance of clinical symptoms of the disease, and assess the rate of SN degeneration. These include positron emission tomography (PET) and single-photon emission computed tomography (SPECT). For example, radiopharmaceutical 18F-fluoroDOPA—a marker of presynaptic dopamine terminals—is an analog of L-DOPA (levodopa) and can interact with the DOPA decarboxylase. PET with 18F-fluoroDOPA, provides a functional approach of pathologies, organs, or tissues where enhanced intracellular transport and decarboxylation of the amino acid dihydroxyphenylalanine is the diagnostic target. This technique allows the activity of DOPA decarboxylase to be measured, and thus assess the metabolism and accumulation of levodopa during the scanning period [37]. PD is characterized by a decrease in the 18F-fluoroDOPA uptake in comparison to age-matched controls [38,39]. PET has allowed calculating the rate of loss of dopaminergic neurons per year. According to various researches, this amount is from 2% to 9% annually [40,41,42]. Thus, the estimated duration of the pre-clinical stage of PD is about seven years [41,43]. PET can also be used for the differential diagnosis of PD, ET, and vascular parkinsonism [41,44]. However, this method is not able to reliably differentiate PD from progressive supranuclear palsy (PSP), multiple system atrophy (MSA), or corticobasal degeneration (CBD). PET imaging allows the promotion of innovative approaches for PD therapy (neurotransplantation, etc.) more effectively since it provides invaluable objective quantitative information about the progression of brain substance damage in dynamics.

Evaluating the state of presynaptic structures can be done using PET with other radiopharmaceuticals, such as [11C]dihydrotetrabenazine (DTBZ). This radioligand allows the tagging of vesicular monoamine carriers. This method can be used to assess the presynaptic status of the nigrostriatal system in PD, including at the premotor stage of the neurodegenerative process [45,46].

Brain SPECT imaging of the dopamine transporter (DAT) with specific radioligands is a sensitive method for examining the integrity of the presynaptic dopaminergic system. This method can reliably distinguish patients with PD from the control group or patients with ET, but it is not possible to reliably differentiate typical and atypical variants of parkinsonism [47,48]. DAT scanning can also be used to detect subclinical dopamine deficiency in the striatum in patients at high risk of developing PD. The limitation of the method is high cost, low availability of equipment, and the use of radioligands [49,50].

MRI-based visualization of nigrosome-1 and neuromelanin can be used as markers for SN assessing in PD. Usually, nigrosome-1 is not detected in patients with PD, i.e., there is no hyperintense signal in the corresponding area of SN. It is suggested that the absence of nigrosome-1 visualization in PD may be associated not only with the loss of dopaminergic neurons but also with the simultaneous process of significant iron accumulation [51,52,53]. The sensitivity of this technique in differentiating PD from the normal and non-degenerative parkinsonism approaches 100%, and the specificity in these cases is 87–100% [52,54,55,56]. Thus, the technique of MRI visualization of nigrosome-1 allows differentiating PD patients from health subjects even at the onset of the disease. However, some cases of atypical parkinsonism (MSA, PSP, and CBD) may be characterized by a similar pattern of the disappearance of the nigrosome-1 to PD [57,58,59].

The neuromelanin-sensitive MRI is another sensitive technique for SN assessment. SN depigmentation is a significant pathological characteristic of PD; it is associated with the loss of neuromelanin, whose paramagnetic properties lead to an increase in the magnetic resonance signal on T1-weighted images. Recent studies have shown that a decrease of neuromelanin in SN in PD can be considered as a potential biomarker of the disease. The neuromelanin-sensitive MRI allows distinguishing PD cases from healthy individuals with high sensitivity and specificity, but where neuromelanin is stable during the disease [60,61,62].

Olfactory involvement is well recognized in PD patients. Despite the good prognostic significance of the marker, the specificity of hyposmia is not high, since it can precede not only PD and dementia with Lewy bodies (DLB), but also Alzheimer’s disease. The decrease in olfaction is explained by pathomorphological studies, which show that olfactory bulbs are affected at the initial stages even before the involvement of the SN in the process of neurodegeneration [63,64]. The relative risk of developing PD among people with hyposmia for three years was 3.9, which is four times less compared to the SNH [10]. This may indicate that olfactometry should not be used as a first-line biomarker, but as a dynamic observation biomarker that reflects the relatively rapid development of motor symptoms [65].

Color visual evoked potentials (VEP) can be used as a method that provides certain diagnostic assistance in patients with PD. The method can be used to diagnose and clarify the nature of visual dysfunction and color perception disorders, and at the earliest stages of the disease. The parameters of color VEP are related to age characteristics, the form of the disease, and the therapy [66,67].

Several studies have shown that about 90% of patients with rapid eye movement (REM) sleep behavior disorder (RBD), confirmed by polysomnography, suffer from various variants of synucleinopathy: PD, dementia with Lewy bodies (DLB), and MSA. On average, the period from the onset of behavior disorders in the REM phase of sleep to PD is about 13 years (in some cases, more than 20–25 years). In the general population the incidence of REM disorders is small, and according to some data, is 1–1.5%. Despite the very high specificity of this marker, its sensitivity for the diagnostics of the prodromal stage of PD is not so high, since only a third of patients with early-stage PD are detected in the REM phase of sleep [68,69].

Electromyography (EMG) study using skin electrodes reveals a change in patients with PD. Changes in EMG can be detected at the subclinical and early stages of PD. In 17.3% and 26.2% of cases of clinically healthy middle-aged and elderly people, respectively, changes are registered, which reflects the presence of hidden extrapyramidal insufficiency and the weakening of inhibitory suprasegmental effects with age. Examination of clinically intact limbs in patients with stage 1 PD using EMG with spectral analysis, revealed changes in 71% of cases in the upper extremities and 58% of cases in the lower extremities. These data are of particular interest as a prospective possibility of using this technique as a tool that facilitates early diagnosis of PD [70,71].

## 3. Metabolomics and Other Omics for PD Diagnostics

It is known that many factors are involved in the pathogenesis of PD, including genetic, age, and environmental factors [20]. The proportion of genetic factors involved in the pathogenesis of PD, and successfully identified using modern high throughput technologies through genome-wide association studies, have dramatically risen to date. At the same time, researchers found a large number of variants of unknown significance. The genes that have been found to potentially cause PD are termed “PARK” and numbered in the order they were identified. To date, 23 PARK genes have been linked to PD. Unfortunately, the linkage or association of some of these genes has not been conclusively confirmed, while variations in some others are considered risk factors. For example, *UCHL1* (PARK5) has been identified in only one family and not replicated since described, and homozygous and compound-heterozygous mutations in *ATP13A2* (PARK9) have been found to cause an atypical form of PD [72]. The first identified PD-related gene was the alpha-synuclein (*SNCA*, PARK1) gene. This protein is considered a key in the pathogenesis of PD since it is the pathological aggregation of alpha-synuclein that is associated with the formation of pathognomonic Lewy bodies in nigral neurons. In addition to alpha-synuclein, Lewy bodies contain proteins involved in the ubiquitin–proteasome system, which is confirmed by the detection of mutations in the E3-ubiquitin ligase (*PRKN*, PARK2) gene. The role of mitochondrial dysfunction in the pathogenesis of PD and associated mutations in the *PRKN* (PARK2), *PINK1* (PARK6), *DJ-1* (PARK7), and *LRRK2* (PARK8) genes is widely discussed [73]. Characteristic mutations, approximate frequency of their occurrence in several populations, and some phenotypic features are described for most of these genes [74,75].

PD also shows abnormalities in the lysosomal protein degradation pathway, which is confirmed by the significance of mutations in the glucocerebrosidase (*GBA*) gene. Homozygosity for mutations in this gene is the basis of Gaucher’s disease, while the heterozygous carrier of mutations in the *GBA* is associated with the development of PD (in these cases, the autosomal dominant inheritance of PD with a very low tolerance of the mutant gene). The association of PD with mutations in the *GBA* highlights the significance of lysosomal autophagy in the molecular mechanisms of the disease [76]. Approximately 7–12% of PD patients have *GBA* mutations, but frequency varies depending on the population. For example the multicentric study of the frequency of *GBA* mutations in an ethnically diverse group of patients with PD has shown that since the carrier frequency of *GBA* mutations is much higher among Ashkenazi Jews, over 15% of Ashkenazi Jewish patients with PD carry the *GBA* mutation [76,77,78]. The overall incidence of *GBA*-associated Parkinson’s disease in the Russian population is 11.6% [79]. The whole-gene sequencing makes it possible to find *GBA* mutations associated with PD. The most frequent *GBA* mutations identified in PD patients are L444P and N370S, characterized by the severe and mild type of Gaucher’s disease, respectively [80,81]. Another two mild *GBA* alterations, which do not in themselves cause Gaucher’s disease but may modify glucocerebrosidase activity, E326K and T369M, still predispose patients to parkinsonism [76]. The meta-analysis of 13 published case-control studies has shown that the *GBA* p.T369M substitution was associated with an increased risk for PD [81].

Even though only 5–10% of all cases of PD are represented by hereditary (monogenic) forms, automated diagnostic panels have already been developed for the differential diagnosis of familial forms of this disease. Nowadays the most widely used methods are massive parallel sequencing and multiplex ligation-dependent probe amplification (MLPA). MLPA is a cheap, simple, rapid, and sensitive tool to detect exon dosage alterations and specific point mutations in selected genes, suitable for developing a PD-MLPA assay [82]. In particular, MRC Holland (Netherlands) offers a set of SALSA MLPA P051-C2, which includes reagents for detecting mutations in the genes of the main proteins involved in the etiopathogenesis of Parkinson’s disease: the protein genes *SNCA* (alpha-synuclein), *PARK2* (parkin), *LRRK2* (dardarin), and *PINK1*. This panel allows studying polymorphisms in candidate genes, presumptively determining the risk of developing PD (*SNCA*, *MAPT*) [83,84]. Nevertheless, use of genetics for PD diagnosis is quite challenging due to the highly polygenic nature of the disease in which common variants confer risk to sporadic PD in the majority of the cases, while hereditary PD cases harbor very rare deleterious variants. Each new genome-wide association study revealed novel variants “associated” with the disease, but frequently they appeared to be specific to studied cohorts and only a few of them could subsequently be replicated in independent studies. Despite genetic forms of PD being rare overall, and mutations diversity being wide overall in the population, they are of major importance for a better understanding of the pathophysiology of the disease and can serve as an excellent human model for the identification of at-risk factors by another omics level.

In addition to gene analysis, approaches related to the study of the transcriptome of PD patients attract much attention [85]. In particular, minimally invasive approaches that are not associated with CSF selection are of great interest. Wang and co-authors proposed a method for determining individual handwriting combinations (*ST13*, *GSK3B*, *HIP*, and *HSPF9*) in the blood cells, whose expression significantly changes in PD patients [86]. In the work of Karlsson and co-authors a biochip was proposed for analyzing the expression of 650 informative genes in the blood of PD patients at different stages of the disease with a sensitivity of 84–88%, which is close to the results of clinical diagnostics [87]. A set of several differently expressed microRNAs in CSF can classify PD and MSA patients from controls with good diagnostic accuracy by evaluating the receiver operating characteristic (ROC-curve). An important diagnostic value is the analysis of the content of that micro-RNAs. The article by C. Starhof and co-authors shows that the analysis of Mir-7-5p, miR-331-5p, and miR-145-5p microRNAs in CFS made it possible to diagnose PD with sensitivity of 88% (compared to the control) [88]. Another study of 35 untreated and 12 treated patients with the sporadic form of PD (Hoehn and Yahr score 1–2) proposed a method for analyzing the expression of micro-RNA levels of *ATP13A2*, *PARK7*, and *ZNF746* in the peripheral blood, which can be suggested also as potential biomarkers of the preclinical stage of PD [89].

Since the key molecular event in PD is the launch of the alpha-synuclein cascade, this protein as well as its metabolites and biochemical partners attract the most attention as potential biomarkers. Recent studies have shown that an increase in the level of oligomeric alpha-synuclein in blood plasma has a high specificity (85%) in clinically diagnosed patients with idiopathic PD, compared to that of the control group [90]. At the same time, this biomarker is typical for the entire group of different synucleinopathies and is not suitable for differentiating individual nosological forms within this group [91]. Perhaps a more promising and specific test will be the analysis of the content of modified forms of alpha-synuclein in physiological fluids—primarily its phosphorylated forms. It is known that 90% of alpha-synuclein deposited in Lewy bodies is phosphorylated. The average level of phosphorylated alpha-synuclein was shown to be significantly higher in cerebrospinal fluid (CSF) and in blood plasma in patients with PD [92,93]. At the same time, the total content of alpha-synuclein in the CSF, on the contrary, underwent a decrease against the background of the development of PD. Thus, a combination of phosphorylated and total alpha-synuclein CSF concentrations can be contributed to distinguish PD patients from MSA and PSP [93].

Tau protein, a component of the neuronal cytoskeleton that polymerizes monomeric tubulin during microtubule assembly, is considered as a possible candidate for the role of a PD biomarker. A decrease of tau protein and its phosphorylated form (p-tau181) in CFS of PD patients was shown [94]. There is an approach that suggests that PD biomarkers should not be considered as individual protein molecules, but rather a combination of two or more proteins involved in the pathogenesis of the disease. Consequently, S. Youness-Mheny and co-authors (2007) suggested considering the ratio of total and phosphorylated tau protein and alpha-synuclein as a biomarker of PD [95]. Another promising diagnostic combination is the ratio of beta-amyloid protein to tau protein. This combination is important for the differential diagnosis of PD with reduced cognitive function and Alzheimer’s disease. It was found that PD is characterized by a decrease in certain isoforms of beta-amyloid (A-beta-40 and A-beta-42), but the concentration of tau protein does not change. Based on the study of CFS samples from a large cohort of PD patients at different stages, as well as healthy and diseased controls, the authors proposed a combination of alpha-synuclein, DJ-1 protein, total tau protein, phosphorylated tau protein, beta-amyloid peptide l-42, Fit3 ligand, and microglial inflammatory mediator fractalkine, as diagnostic markers not just for PD diagnosis but for differentiation from MSA patients, and correlation with disease severity and progression [96]. For a long time, the DJ-1 protein was considered as a candidate for the role of a biomarker in PD, since mutations in the gene encoding this protein (*PARK7*) cause an autosomal recessive form of PD. However, conflicting results were obtained for this protein in patients with PD, both a decrease and an increase in the concentration of it in both blood plasma and CFS were recorded [97]. As a result, Lin et al. proposed a method for the direct determination of one of the DJ-1 isoforms (7DJ-1, with posttranslational modification of 4-hydroxy-2-nonenal), whose plasma level significantly increase in late-stage PD [98].

Important diagnostic and prognostic markers of PD development are neuron-specific autoantibodies. Based on the analysis of the spectrum of 10 antibodies contained in the blood serum samples of 29 PD, 50 Alzheimer’s disease, and 40 control patients, a PD test has been developed with a sensitivity of 93% and specificity of 100%. The most diagnostically significant antibodies included in this test are antibodies to the intercellular adhesion molecule 4 (ICAM4), antibodies to pentatricopeptide with repeated domain 2 (PTCD2), to myotilin (MYOT), and fibronectin 1 (FN1) [99]. Another group of authors obtained data on two new protein biomarkers of Parkinson’s disease—mitochondrial ribosome recycling factor (MRRF) and ribosomal protein S18 (RPS18), the increase of which is particularly relevant in determining the prodromal stage of Parkinson’s disease [100]. Proteomics research over the past decade has significantly expanded the range of biomarker candidate proteins. Marker values of BDNF, NGF and IGF-1 neurotrophins, haptoglobin, beta-2 microglobulin, vitamin D binding protein, pro-inflammatory cytokines (chemokine CXCL8), epidermal growth factor, and apolipoprotein A1 are actively studied. The increased level of the latter directly correlates with the age of PD onset, which allows considering this protein as a predictor of the risk of PD development [101,102].

Although a range of biomarkers derived from clinical, neuroimaging, genetic, and proteomics PD studies have been proposed, there are no laboratory tests for its early diagnostics. One of the promising approaches for developing methods for PD’s early diagnostics is metabolomics since it is aimed at a detailed description of the composition of biological objects and biochemical reactions in which low-molecular substances participate as substrates, intermediates, and reaction products [103]. Initially, metabolomics methods were mainly used to study PD for analysis of individual metabolites of interest (catecholamines, amino acids, purines, and urates) in biological fluids such as blood, CFS, and urine [104,105,106]. Thus, the UPLC-MS/MS quantitative analysis of plasmatic circulating trace amines in de novo and treated PD patients has revealed that while tyramine behaves as a putative biomarker for early-stage PD (AUC (area under ROC curve) = 0.90) tyramine, norepinephrine, and tyrosine appear to act as biomarkers of disease progression (AUC > 0.75) [107]. Using the LC-electrochemistry array (LCECA) based targeted approach, a significant reduction of catecholamines including homovanillic acid (HVA), dihydroxyphenylacetic acid (DOPAC), dopamine, and dihydroxyphenylglycol (DHPG), have been reported in CFS of PD patients especially with recent onset of parkinsonism [106].

In the last decade, untargeted metabolomics analysis (profiling) of biological fluids has been used to search for new biomarkers especially in CFS and blood. For example, in the study by Bogdanov et al. metabolomics analysis of blood plasma sample profiles made it possible to clearly differentiate patients with PD from healthy donors. In particular, differences were found in the levels of uric acid and glutathione [108]. Many of the studies report alterations in alanine, branched-chain amino acids, and fatty acid metabolism, all pointing to mitochondrial dysfunction in PD. Aromatic amino acids (phenylalanine, tyrosine, tryptophan) and purine metabolism (uric acid) are also altered in most metabolite profiling studies of PD [109]. One of the technologies most suitable for clinical use is metabolomic profiling by direct injection of a low-molecular fraction of a blood sample into an electrospray ionization source of a mass spectrometer. This method has shown outstanding results for diagnostics of many socially significant diseases and does not require complex sample preparation and any preliminary separation, which simplifies the adaptation of this metabolomic analysis for implementation in clinical diagnostic practice [110,111,112,113,114,115,116,117].

In the study by Balashova et al. of blood plasma samples of PD and control patients by direct injection mass spectrometry analysis, the metabolome signature with high PD’s diagnostic significance (accuracy—94%, sensitivity—94%, and specificity—95%) was detected [118]. The main metabolites of the signature were associated with a high level of degradation of the amino acid lysine, which is confirmed by the presence of the well-known phenomenon of a decrease in the level of lysine in the blood plasma of patients with PD. The level of phospholipids in the blood of PD patients was also reduced. Other metabolites, such as, for example, hydroxyisovalerylcarnitine, on the contrary, were increased, which can serve as a marker of fatty acid oxidation. This process can be a result of oxidative stress and is typical for many neurodegenerative disorders, including PD. 3-hydroxyisovalerylcarnitine is associated with acylcarnitines, as acylcarnitine analysis can be used to diagnose many fatty acid oxidation disorders. Histamine was also increased in samples of PD patients, which was confirmed by another study that found abnormal levels of histamine in the brain in PD [119]. Putrescine is associated with polyamines and is produced when amino acids are broken down in living and dead organisms. It is neurotoxic, and its use leads to shocking behavior and motor disorders. Therefore, it seems reasonable that an increased level of putrescine may be responsible for some clinical manifestations of PD. Another metabolite found in the study was asymmetric dimethylarginine—a chemical formed in the blood plasma during protein methylation. This reaction is catalyzed by an enzyme from the N-methyltransferase family. Their increased activity may lead to a high level of asymmetric dimethylarginine. Thus, the identified PD’s metabolome signature reflects most characteristic changes in the plasma metabolome, well confirmed by previously published works in this field and can be seen as a multivariate biomarker with improved diagnostic capabilities when compared to modern approaches. At the same time, to implement this signature into clinical practice as a PD biomarker, additional verification of this signature on a larger population of PD patients is required.

An important advantage of the metabolomic approach for PD’s diagnostics and prognosis is the ability to evaluate pathochemical processes occurring at different stages of disease development, based on which it is possible to develop new strategies of pathogenetic therapy for the disease, in particular, by correcting aberrant metabolic processes associated, for example, with the accumulation of neurotoxic chemical agents. Additionally, the use of the metabolomics approach opens a wide range of possibilities for PD’s drug therapy personalization, taking into account the individual metabolic status of a particular patient [120,121,122,123]. For example, the most widely used drug in PD therapy, levodopa, as a precursor of dopamine in the central nervous system, can be metabolized in peripheral tissues and organs to oxymethylated derivative-3-O-methyl-DOPA (3-OMD), which is a functional antagonist of levodopa [124]. Other metabolites of levodopa are products of its oxidation, such as various semiquinones as well as neurotoxic 5-S-cysteinyldopa, can potentially contribute to the development of oxidative stress in PD. The metabolomics technologies can be used for the timely detection of pathological accumulation of various aberrant metabolites of levodopa, which will minimize the risk of undesirable drug reactions during substitution dopaminergic therapy of PD.

However, while recognizing the promise of using molecular and biochemical biomarkers in PD, it should be noted that these approaches are currently primarily research-based and have not yet been included in everyday clinical practice (Appendix A, Table A1). Furthermore, it is important to remember that existing molecular biomarkers are only effective in combination with other diagnostics methods and should be carefully considered in the total clinical picture of the disease including the medical history of the patient.

## 4. Conclusions

Summarizing the above, it can be said that the creation of metabolomics systems for analyzing the development of the pathological process in PD will not only allow early detection of the disease but will also open the perspective of creating methods for correction of biochemical disorders occurring at the premotor stage of the disease. Thoughtful integration of the metabolomics findings with multidimensional data from genomics, transcriptomics, and proteomics studies in combination with available clinical tests can provide comprehensive system-level insight into the PD development mechanisms and drug therapy.

## Figures and Tables

**Table 1:** The criteria for Parkinson’s disease (PD) diagnostics according to the Movement Disorder Society guidelines.

Category	Diagnostic Features
Main Parkinsonism Diagnostics Criteria	Bradykinesia (with speed and amplitude decrement) in combination with rest tremor, or/and muscle rigidity.
Supportive Criteria	An adequate response to dopaminergic therapy. Levodopa peak–dose dyskinesia. Limb rest tremor. Hyposmia confirmed by the University of Pennsylvania Smell Identification Test (UPSIT), and/or progression of myocardial sympathetic denervation assessed by metaiodobenzylguanidine (MIBG) myocardial scintigraphy.
Absolute Exclusion Criteria	Cerebellar abnormalities. Downward gaze paresis or slowing of vertical saccades. A behavioral variant of frontotemporal dementia or primary progressive aphasia during the first 5 years of the disease. “Lower body parkinsonism” for more than 3 years. Treatment with neuroleptics during the sufficient period and in a dose that can cause parkinsonism. Absence of observable response to levodopa therapy in high doses. Unequivocal cortical sensory loss (i.e., graphesthesia or stereognosis with intact primary sensory modalities), clear limb ideomotor apraxia, or progressive aphasia. Normal functional neuroimaging of the presynaptic dopaminergic system confirmed by single-photon emission or positron emission tomography. The presence of another disease that can cause Parkinson’s syndrome.
Red Flags	Rapid progression of gait impairment requiring regular use of wheelchair within 5 years of onset. Absence of progression of motor symptoms or signs over 5 or more years unless related to treatment. Early bulbar dysfunction: severe dysphonia or dysarthria or severe dysphagia within first 5 years. Inspiratory respiratory dysfunction: either diurnal or nocturnal inspiratory stridor or frequent inspiratory sighs. Severe autonomic failure in the first 5 years of the disease. E.g., (a) severe orthostatic decrease of blood pressure within 3 min of standing by at least 30 mm Hg systolic or 15 mm Hg diastolic or (b) severe urinary retention or urinary incontinence in the first 5 years of the disease. Recurrent (> 1/year) falls because of impaired balance within 3 years of disease. Disproportionate anterocollis (dystonic) or contractures of hand or feet within the first 10 years. Absence of any of the common nonmotor features of disease despite 5 years of the disease duration including sleep disorders, autonomic dysfunction, hyposmia, neuropsychiatric disorders (depression, anxiety, or hallucinations). Otherwise-unexplained pyramidal tract signs, defined as pyramidal weakness or clear pathologic hyperreflexia. Bilateral symmetric parkinsonism. The patient or caregiver reports bilateral symptom onset with no side predominance and no side predominance is observed on objective examination.

## Appendix A

**Table A1:** Summary of the main clinical and promising omics-based tests for PD diagnostics.

Technique	Summary
**Clinical Tests**	
Pharmacological test using levodopa and/or dopamine agonists for the dopaminergic treatment.	Performed for confirmation of the diagnosis of PD and differentiating PD from the other parkinsonian syndromes [22,23,24].
Imaging techniques:	
1. Transcranial sonography (TCS) for investigation of the morphology of substantia nigra (SN) and assessing of substantia nigra hyperechogenicity (SNH) due to its increased iron content.	Used for the diagnosis of PD at any stage and establishing a predisposition to the development of PD in individuals before the onset of motor symptoms of the disease; also can be used for differential diagnosis with essential tremor (ET) and atypical parkinsonism [25,26,27,28,35,36].
2. Positron emission tomography (PET) for measuring the activity of DOPA decarboxylase and thus assessing the metabolism and accumulation of levodopa during the scanning period.	Used for the differential diagnosis of PD, ET, and vascular parkinsonism, but not able to reliably differentiate PD from progressive supranuclear palsy (PSP), multiple system atrophy (MSA), or corticobasal degeneration (CBD) [37,38,39,40,41,42,43,44].
3. Single-photon emission computed tomography (SPECT) for brain imaging of the dopamine transporter (DAT) with specific radioligands.	Used for distinguishing patients with PD from the control group or patients with ET, but it is not possible to reliably differentiate typical and atypical variants of parkinsonism [47,48,49,50].
4. Magnetic resonance imaging (MRI) for visualization of nigrosome-1 and neuromelanin for SN assessment.	Used for differentiating PD patients from healthy subjects, but not able to differentiate from some cases of atypical parkinsonism (MSA, PSP, and CBD) [51,52,53,54,55,56,57,58,59,60,61,62].
Olfactometry for hyposmia assessment.	Despite the good prognostic significance of the marker, the specificity of hyposmia is not high, since it can precede not only PD and dementia with Lewy bodies (DLB) but also Alzheimer’s disease [63,64,65].
Color visual evoked potentials (VEP) for diagnosing and clarifying the nature of visual dysfunction and color perception disorders.	The marker is related to age characteristics, the form of the PD, and the therapy [66,67].
Polysomnography for rapid eye movement (REM) sleep behavior disorder (RBD) assessment.	Performed for diagnostics various variants of synucleinopathy: PD, DLB, MSA, but not so sensitive for the prodromal stage of PD [68,69].
Electromyography (EMG) for the examination of clinically intact limbs in patients using skin electrodes.	EMG changes have been revealed in 71% of cases in the upper extremities and 58% of cases in the lower extremities in patients with early-stage PD and can facilitate early diagnosis of PD [70,71].
**Omics-Based Tests**	
Genomics:	
1. Identification of mutations in the alpha-synuclein (*SNCA*) gene (PARK1).	Duplications, triplications, or point mutation in *SNCA* cause autosomal dominant forms of PD and are the basis of the risk of developing sporadic PD [73,74,75].
2. Identification of mutations in the E3-ubiquitin ligase (*PRKN*) gene (PARK2).	The most common cause of autosomal recessive early-onset PD with frequency estimations ranging from 4.6% to 10.5%, depending on the population [73,74,75].
3. Identification of mutations in the phosphatase and tensin homolog (*PTEN*)-induced putative kinase 1 (*PINK1*) gene (PARK6).	The second most common cause of autosomal recessive early-onset PD with frequency in the range of 1–9%, depending on the population [73,74,75].
4. Identification of mutations in the *DJ-1* gene (PARK7).	The third most common cause of autosomal recessive early-onset PD with frequency in the range of 1–2% of cases [73,74,75].
5. Identification of mutations in the *LRRK2* gene (PARK8).	The most frequent known cause of late-onset autosomal-dominant and sporadic PD, with a mutation frequency ranging from 2% to 40% in different populations [73,74,75].
6. Identification of mutations in the glucocerebrosidase (*GBA*) gene.	The heterozygous carrier of mutations in the *GBA* gene is associated with the development of PD with frequency in the range of 7–12% depending on the population [76].
Transcriptomics:	
1. Quantitative PCR assays analysis of previously identified RNA biomarkers—*PTPN1*, *COPZ1*, *FAXDC2*, *SLC14A1s,* and *NAMPT* in the blood.	Linear discriminant analysis showed that *COPZ1* and *PTPN1* distinguished PD from PSP patients with 62.5% accuracy. Five biomarkers, *PTPN1*, *COPZ1*, *FAXDC2*, *SLC14A1s,* and *NAMPT* were useful for distinguishing PSP from controls with 69% accuracy [85].
2. Quantitative PCR analysis of expression levels of the CFS- microRNAs: Mir-7-5p, miR-331-5p, miR-145-5p, miR-9-3p, and miR-106b-5p.	Level of the set of mir-7-5p, miR-331-5p, and miR-145-5p discriminated PD from controls with accuracy of 88%; level of the set of miR-9-3p and miR-106b-5p distinguished PD from MSA with accuracy of 73%; and level of miR-106b-5p provided the best discrimination between PD and PSP with accuracy of 85% [88].
3. Analysis of the mRNA levels of *ATP13A2*, *PARK2*, *PARK7*, *PINK1*, *LRRK2*, *SNCA*, *ALDH1A1*, *PDHB*, *PPARGC1A*, and *ZNF746* genes in the peripheral blood.	A statistically significant increase in the mRNA levels of *ATP13A2*, *PARK7*, and *ZNF746* genes was observed in the group of untreated patients with PD but not in the neurological disease control group [89].
Proteomics:	
1. Immunoassay analysis of alpha-synuclein and phosphorylated alpha-synuclein (PS-129) in CFS and blood plasma.	A combination of PS-129 and total alpha-synuclein in CFS performed better than PS-129 alone in detection of PD, MSA, and PSP patients versus healthy controls with sensitivities of 61%, 75%, and 67%, respectively. The sensitivities among the three different parkinsonian disease groups were: PD versus MSA patients, 40%; PD versus PSP patients, 72%; and MSA versus PSP patients, 63%. The diagnostic values (specificity) were PD versus controls, 64%; MSA versus controls, 73%; PSP versus controls, 55%; PD versus MSA, 63%; PD versus PSP, 63%; and MSA versus PSP, 67% [92,93].
2. Immunoassay or enzyme-linked immunosorbent assay measuring of total and phosphorylated tau protein, beta-amyloid peptide l-42, and alpha-synuclein in CFS.	Slightly, but significantly lower levels of these proteins were seen in subjects with PD compared with healthy controls [94].
3. Measuring using Luminex assays of alpha-synuclein, DJ-1 protein, total and phosphorylated tau protein, beta-amyloid peptide l-42, Fit3 ligand, and microglial inflammatory mediator fractalkine in CFS.	A combination of DJ-1 plus Flt3 ligand differentiates PD from control with sensitivity 94% and specificity 60%. With CSF Flt3 ligand, MSA patients were differentiated from PD patients with sensitivity 99% and specificity 95%, and high sensitivity (90%) and a reasonable specificity (71%) could also be achieved using a combination of alpha-synuclein and ratio of p-tau and t-tau [96].
4. Analysis of expression of potential biomarker autoantibodies in the blood serum using human protein microarrays, each containing 9486 native antigens.	10 selected autoantibodies with a different expression, including antibodies to intercellular adhesion molecule 4 (ICAM4), pentatricopeptide with repeated domain 2 (PTCD2), myotilin (MYOT), and fibronectin 1 (FN1), effectively differentiated PD from control with a sensitivity of 93.1% and specificity of 100% and also distinguished PD from Alzheimer’s disease with accuracy of 86% [99].
5. Identification of distinct blood protein autoantibody biomarkers of early-stage PD by using the Gene Expression Omnibus database.	Two biomarkers, mitochondrial ribosome recycling factor (MRRF) and ribosomal protein S18 (RPS18), distinguished the early-stage PD samples from the control samples and can be regarded as high-confidence distinct protein autoantibody biomarkers of early-stage PD [100].
Metabolomics:	
1. Quantification of plasmatic TAs, and the catecholamines and indolamines pertaining to the same biochemical pathways using an ultra-performance chromatography mass spectrometry (UPLC-MS/MS) method.	Tyramine has been suggested as a promising marker for assessing the disease at an early stage of PD (AUC = 0.90). Tyramine, norepinephrine, and tyrosine showed the possibility that these compounds behave as promising markers for the progression of the disease (AUC > 0.75) [107].
2. Measuring of CSF and plasma levels of catechols including dopamine, norepinephrine, and their main respective neuronal metabolites dihydroxyphenylacetic acid and dihydroxyphenylglycol.	CSF level of dihydroxyphenylacetic acid was 100% sensitive at 89% specificity in separating patients with recent onset of PD from controls but was of no value in differentiating PD from MSA [106].
3. Metabolomic profiling of blood plasma samples using high-performance liquid chromatography coupled with electrochemical coulometric array detection (LCECA).	Obtained blood plasma samples metabolomics profiles made it possible to clearly differentiate patients with PD from healthy donors. In particular, uric acid was significantly reduced while glutathione was significantly increased in PD patients [108].
4. Metabolomic profiling of blood plasma samples by direct injection mass spectrometry.	The metabolome signature of 21 metabolite ions, including lysine, phospholipids, hydroxyisovalerylcarnitine, histamine, putrescine, and asymmetric dimethylarginine, with high PD’s diagnostic significance (accuracy—94%, sensitivity—94%, specificity—95%), was detected [118].

## References

[1] Tysnes O.B., Storstein A. (2017). Epidemiology of Parkinson’s disease. J. Neural Transm..

[2] Kalia L.V., Lang A.E. (2015). Parkinson’s disease. Lancet.

[3] Reich S.G., Savitt J.M. (2019). Parkinson’s Disease. Med. Clin. North Am..

[4] Deutschländer A.B., Ross O.A., Dickson D.W., Wszolek Z.K. (2018). Atypical parkinsonian syndromes: A general neurologist’s perspective. Eur. J. Neurol..

[5] Williams D.R., Litvan I. (2013). Parkinsonian syndromes. Contin. Lifelong Learn. Neurol..

[6] Postuma R.B., Berg D., Stern M., Poewe W., Olanow C.W., Oertel W., Obeso J., Marek K., Litvan I., Lang A.E. (2015). MDS clinical diagnostic criteria for Parkinson’s disease. Mov. Disord..

[7] Postuma R.B., Poewe W., Litvan I., Lewis S., Lang A.E., Halliday G., Goetz C.G., Chan P., Slow E., Seppi K. (2018). Validation of the MDS clinical diagnostic criteria for Parkinson’s disease. Mov. Disord..

[8] Heinzel S., Berg D., Gasser T., Chen H., Yao C., Postuma R.B. (2019). Update of the MDS research criteria for prodromal Parkinson’s disease. Mov. Disord..

[9] Berardelli A., Wenning G.K., Antonini A., Berg D., Bloem B.R., Bonifati V., Brooks D., Burn D.J., Colosimo C., Fanciulli A. (2013). EFNS/MDS-ES recommendations for the diagnosis of Parkinson’s disease. Eur. J. Neurol..

[10] Berg D., Marek K., Ross G.W., Poewe W. (2012). Defining at-risk populations for Parkinson’s disease: Lessons from ongoing studies. Mov. Disord..

[11] Sui X., Zhou C., Li J., Chen L., Yang X., Li F. (2019). Hyposmia as a predictive marker of Parkinson’s disease: A systematic review and meta-analysis. Biomed Res. Int..

[12] Yoneyama N., Watanabe H., Kawabata K., Bagarinao E., Hara K., Tsuboi T., Tanaka Y., Ohdake R., Imai K., Masuda M. (2018). Severe hyposmia and aberrant functional connectivity in cognitively normal Parkinson’s disease. PLoS ONE.

[13] Armstrong R., Kergoat H. (2015). Oculo-visual changes and clinical considerations affecting older patients with dementia. Ophthalmic Physiol. Opt..

[14] Ekker M.S., Janssen S., Seppi K., Poewe W., de Vries N.M., Theelen T., Nonnekes J., Bloem B.R. (2017). Ocular and visual disorders in Parkinson’s disease: Common but frequently overlooked. Park. Relat. Disord..

[15] Borm C.D.J.M., Smilowska K., De Vries N.M., Bloem B.R., Theelen T. (2019). How i do it: The Neuro-Ophthalmological Assessment in Parkinson’s Disease. J. Parkinsons Dis..

[16] Kilzheimer A., Hentrich T., Burkhardt S., Schulze-Hentrich J.M. (2019). The Challenge and Opportunity to Diagnose Parkinson’s Disease in Midlife. Front. Neurol..

[17] Sveinbjornsdottir S. (2016). The clinical symptoms of Parkinson’s disease. J. Neurochem..

[18] Lang A.E. (2011). A critical appraisal of the premotor symptoms of Parkinson’s disease: Potential usefulness in early diagnosis and design of neuroprotective trials. Mov. Disord..

[19] Berg D., Postuma R.B., Adler C.H., Bloem B.R., Chan P., Dubois B., Gasser T., Goetz C.G., Halliday G., Joseph L. (2015). MDS research criteria for prodromal Parkinson’s disease. Mov. Disord..

[20] Kouli A., Torsney K.M., Kuan W.-L., Stoker T.B., Greenland J.C. (2018). Parkinson’s Disease: Etiology, Neuropathology, and Pathogenesis. Parkinson’s Disease: Pathogenesis and Clinical Aspects.

[21] Noyce A.J., Bestwick J.P., Silveira-Moriyama L., Hawkes C.H., Giovannoni G., Lees A.J., Schrag A. (2012). Meta-analysis of early nonmotor features and risk factors for Parkinson disease. Ann. Neurol..

[22] Rossi P., Colosimo C., Moro E., Tonali P., Albanese A. (2000). Acute challenge with apomorphine and levodopa in parkinsonism. Eur. Neurol..

[23] Merello M., Nouzeilles M.I., Arce G.P., Leiguarda R. (2002). Accuracy of acute levodopa challenge for clinical prediction of sustained long-term levodopa response as a major criterion for idiopathic Parkinson’s disease diagnosis. Mov. Disord..

[24] Schade S., Sixel-Döring F., Ebentheuer J., Schulz X., Trenkwalder C., Mollenhauer B. (2017). Acute Levodopa Challenge Test in Patients with de novo Parkinson’s Disease: Data from the DeNoPa Cohort. Mov. Disord. Clin. Pract..

[25] Berg D., Roggendorf W., Schröder U., Klein R., Tatschner T., Benz P., Tucha O., Preier M., Lange K.W., Reiners K. (2002). Echogenicity of the substantia nigra: Association with increased iron content and marker for susceptibility to nigrostriatal injury. Arch. Neurol..

[26] Berg D., Godau J., Walter U. (2008). Transcranial sonography in movement disorders. Lancet Neurol..

[27] Prestel J., Schweitzer K.J., Hofer A., Gasser T., Berg D. (2006). Predictive value of transcranial sonography in the diagnosis of Parkinson’s disease. Mov. Disord..

[28] Shafieesabet A., Fereshtehnejad S.M., Shafieesabet A., Delbari A., Baradaran H.R., Postuma R.B., Lökk J. (2017). Hyperechogenicity of substantia nigra for differential diagnosis of Parkinson’s disease: A meta-analysis. Park. Relat. Disord..

[29] Yilmaz R., Berg D. (2018). Transcranial B-Mode Sonography in Movement Disorders. Int. Rev. Neurobiol..

[30] Li X., Feng T., Ouyang Q.H., Zhang H.X., Li F.F. (2013). Comparative study on the diagnostic value of positron emission tomography and transcranial sonography in the diagnosis of Parkinson disease. Natl. Med. J. China.

[31] Toomsoo T., Liepelt-Scarfone I., Kerner R., Kadastik-Eerme L., Asser T., Rubanovits I., Berg D., Taba P. (2016). Substantia Nigra Hyperechogenicity: Validation of Transcranial Sonography for Parkinson Disease Diagnosis in a Large Estonian Cohort. J. Ultrasound Med..

[32] Prati P., Bignamini A., Coppo L., Naldi A., Comi C., Cantello R., Gusmaroli G., Walter U. (2017). The measuring of substantia nigra hyperechogenicity in an Italian cohort of Parkinson disease patients: A case/control study (NOBIS Study). J. Neural Transm..

[33] Berg D., Merz B., Reiners K., Naumann M., Becker G. (2005). Five-year follow-up study of hyperechogenicity of the substantia nigra in Parkinson’s disease. Mov. Disord..

[34] Behnke S., Runkel A., Kassar H.A.S., Ortmann M., Guidez D., Dillmann U., Fassbender K., Spiegel J. (2013). Long-term course of substantia nigra hyperechogenicity in Parkinson’s disease. Mov. Disord..

[35] Berg D., Godau J., Seppi K., Behnke S., Liepelt-Scarfone I., Lerche S., Stockner H., Gaenslen A., Mahlknecht P., Huber H. (2013). The PRIPS study: Screening battery for subjects at risk for Parkinson’s disease. Eur. J. Neurol..

[36] Berg D., Behnke S., Walter U. (2006). Application of transcranial sonography in extrapyramidal disorders: Updated recommendations. Ultraschall der Med..

[37] Moore R.Y., Whone A.L., McGowan S., Brooks D.J. (2003). Monoamine neuron innervation of the normal human brain: An 18F-DOPA PET study. Brain Res..

[38] Morrish P.K., Sawle G.V., Brooks D.J. (1996). An [18F]dopa-PET and clinical study of the rate of progression in Parkinson’s disease. Brain.

[39] Pikstra A.R.A., Van Der Hoorn A., Leenders K.L., De Jong B.M. (2016). Relation of 18-F-Dopa PET with hypokinesia-rigidity, tremor and freezing in Parkinson’s disease. NeuroImage Clin..

[40] Morrish P.K., Sawle G.V., Brooks D.j. (1995). Clinical and [18F]dopa PET findings in early Parkinson’s disease. J. Neurol. Neurosurg. Psychiatry.

[41] Brooks D.J. (2000). Morphological and functional imaging studies on the diagnosis and progression of Parkinson’s disease. J. Neurol. Suppl..

[42] Whone A.L., Watts R.L., Stoessl A.J., Davis M., Reske S., Nahmias C., Lang A.E., Rascol O., Ribeiro M.J., Remy P. (2003). Slower progression of Parkinson’s disease with ropinirole versus levodopa: The REAL-PET study. Ann. Neurol..

[43] Brooks D.J. (2012). Parkinson’s disease: Diagnosis. Park. Relat. Disord..

[44] Brooks D.J., Playford E.D., Ibanez V., Sawle G.V., Thompson P.D., Findley C.W., Marsden C.D. (1992). Isolated tremor and disruption of the nigrostriatal dopaminergic system: An 18F-dopa PET study. Neurology.

[45] Stoessl A.J. (2007). Positron emission tomography in premotor Parkinson’s disease. Park. Relat. Disord..

[46] de Natale E.R., Niccolini F., Wilson H., Politis M. (2018). Molecular Imaging of the Dopaminergic System in Idiopathic Parkinson’s Disease. Int. Rev. Neurobiol..

[47] Ling H., Lees A.J. (2010). How Can Neuroimaging Help in the Diagnosis of Movement Disorders?. Neuroimaging Clin. N. Am..

[48] Politis M. (2014). Neuroimaging in Parkinson disease: From research setting to clinical practice. Nat. Rev. Neurol..

[49] Shih M.C., Hoexter M.Q., de Andrade L.A.F., Bressan R.A. (2006). Parkinson’s disease and dopamine transporter neuroimaging—A critical review. Sao Paulo Med. J..

[50] Armstrong M.J., Okun M.S. (2020). Diagnosis and Treatment of Parkinson Disease: A Review. JAMA J. Am. Med. Assoc..

[51] Lehéricy S., Bardinet E., Poupon C., Vidailhet M., François C. (2014). 7 tesla magnetic resonance imaging: A closer look at substantia nigra anatomy in Parkinson’s disease. Mov. Disord..

[52] Noh Y., Sung Y.H., Lee J., Kim E.Y. (2015). Nigrosome 1 detection at 3T MRI for the diagnosis of early-stage idiopathic Parkinson disease: Assessment of diagnostic accuracy and agreement on imaging asymmetry and clinical laterality. Am. J. Neuroradiol..

[53] Schwarz S.T., Xing Y., Naidu S., Birchall J., Skelly R., Perkins A., Evans J., Sare G., Martin-Bastida A., Bajaj N. (2017). Protocol of a single group prospective observational study on the diagnostic value of 3T susceptibility weighted MRI of nigrosome-1 in patients with parkinsonian symptoms: The N3 i PD study (nigrosomal i ron i maging i n Parkinson’s disease). BMJ Open.

[54] Cosottini M., Frosini D., Pesaresi I., Costagli M., Biagi L., Ceravolo R., Bonuccelli U., Tosetti M. (2014). MR imaging of the substantia nigra at 7 T enables diagnosis of parkinson disease. Radiology.

[55] Gao P., Zhou P.Y., Li G., Zhang G.B., Wang P.Q., Liu J.Z., Xu F., Yang F., Wu X.X. (2015). Visualization of nigrosomes-1 in 3T MR susceptibility weighted imaging and its absence in diagnosing Parkinson’s disease. Eur. Rev. Med. Pharmacol. Sci..

[56] Nam Y., Gho S.M., Kim D.H., Kim E.Y., Lee J. (2017). Imaging of nigrosome 1 in substantia nigra at 3T using multiecho susceptibility map-weighted imaging (SMWI). J. Magn. Reson. Imaging.

[57] Reiter E., Mueller C., Pinter B., Krismer F., Scherfler C., Esterhammer R., Kremser C., Schocke M., Wenning G.K., Poewe W. (2015). Dorsolateral nigral hyperintensity on 3.0T susceptibility-weighted imaging in neurodegenerative Parkinsonism. Mov. Disord..

[58] Pavese N., Tai Y.F. (2018). Nigrosome Imaging and Neuromelanin Sensitive MRI in Diagnostic Evaluation of Parkinsonism. Mov. Disord. Clin. Pract..

[59] Jin L., Wang J., Wang C., Lian D., Zhou Y., Zhang Y., Lv M., Li Y., Huang Z., Cheng X. (2019). Combined Visualization of Nigrosome-1 and Neuromelanin in the Substantia Nigra Using 3T MRI for the Differential Diagnosis of Essential Tremor and de novo Parkinson’s Disease. Front. Neurol..

[60] Saeed U., Compagnone J., Aviv R.I., Strafella A.P., Black S.E., Lang A.E., Masellis M. (2017). Imaging biomarkers in Parkinson’s disease and Parkinsonian syndromes: Current and emerging concepts. Transl. Neurodegener..

[61] Xing Y., Sapuan A., Dineen R.A., Auer D.P. (2018). Life span pigmentation changes of the substantia nigra detected by neuromelanin-sensitive MRI. Mov. Disord..

[62] Huddleston D.E., Langley J., Dusek P., He N., Faraco C.C., Crosson B., Factor S., Hu X.P. (2018). Imaging Parkinsonian Pathology in Substantia Nigra with MRI. Curr. Radiol. Rep..

[63] Kawase Y., Hasegawa K., Kawashima N., Horiuchi E., Ikeda K. (2010). Olfactory dysfunction in Parkinson’s disease: Benefits of quantitative odorant examination. Int. J. Gen. Med..

[64] Passali G.C., Bove F., Vargiu L., Bentivoglio A.R., Anzivino R., De Corso E., Galli J., Rigante M., Pandolfini M., Sergi B. (2017). New olfactometric findings in Parkinson’s disease. Clin. Otolaryngol..

[65] Xiao Q., Chen S., Le W. (2014). Hyposmia: A possible biomarker of Parkinson’s disease. Neurosci. Bull..

[66] Quagliato L.B., Domingues C., Quagliato E.M.A.B., de Abreu E.B., Kara-Junior N. (2014). Applications of visual evoked potentials and fourier-domain optical coherence tomography in Parkinson’s disease: A controlled study. Arq. Bras. Oftalmol..

[67] He S.B., Liu C.Y., Chen L.D., Ye Z.N., Zhang Y.P., Tang W.G., Wang B.D., Gao X. (2018). Meta-Analysis of Visual Evoked Potential and Parkinson’s Disease. Parkinsons. Dis..

[68] Postuma R.B., Berg D. (2016). Advances in markers of prodromal Parkinson disease. Nat. Rev. Neurol..

[69] Fereshtehnejad S.M., Montplaisir J.Y., Pelletier A., Gagnon J.F., Berg D., Postuma R.B. (2017). Validation of the MDS research criteria for prodromal Parkinson’s disease: Longitudinal assessment in a REM sleep behavior disorder (RBD) cohort. Mov. Disord..

[70] Oung Q.W., Muthusamy H., Lee H.L., Basah S.N., Yaacob S., Sarillee M., Lee C.H. (2015). Technologies for assessment of motor disorders in Parkinson’s Disease: A review. Sensors.

[71] Hasan H., Athauda D.S., Foltynie T., Noyce A.J. (2017). Technologies Assessing Limb Bradykinesia in Parkinson’s Disease. J. Parkinsons. Dis..

[72] Klein C., Westenberger A. (2012). Genetics of Parkinson’s disease. Cold Spring Harb. Perspect. Med..

[73] Lill C.M. (2016). Genetics of Parkinson’s disease. Mol. Cell. Probes.

[74] Nalls M.A., Blauwendraat C., Vallerga C.L., Heilbron K., Bandres-Ciga S., Chang D., Tan M., Kia D.A., Noyce A.J., Xue A. (2019). Identification of novel risk loci, causal insights, and heritable risk for Parkinson’s disease: A meta-analysis of genome-wide association studies. Lancet Neurol..

[75] Reed X., Bandrés-Ciga S., Blauwendraat C., Cookson M.R. (2019). The role of monogenic genes in idiopathic Parkinson’s disease. Neurobiol. Dis..

[76] Do J., McKinney C., Sharma P., Sidransky E. (2019). Glucocerebrosidase and its relevance to Parkinson disease. Mol. Neurodegener..

[77] Sidransky E., Nalls M.A., Aasly J.O., Aharon-Peretz J., Annesi G., Barbosa E.R., Bar-Shira A., Berg D., Bras J., Brice A. (2009). Multicenter analysis of glucocerebrosidase mutations in Parkinson’s disease. N. Engl. J. Med..

[78] Gan-Or Z., Amshalom I., Kilarski L.L., Bar-Shira A., Gana-Weisz M., Mirelman A., Marder K., Bressman S., Giladi N., Orr-Urtreger A. (2015). Differential effects of severe vs mild GBA mutations on Parkinson disease. Neurology.

[79] Shulskaya M.V., Alieva A.K., Vlasov I.N., Zyrin V.V., Fedotova E.Y., Abramycheva N.Y., Usenko T.S., Yakimovsky A.F., Emelyanov A.K., Pchelina S.N. (2018). Whole-exome sequencing in searching for new variants associated with the development of Parkinson’s disease. Front. Aging Neurosci..

[80] Mao X., Wang T., Peng R., Chang X., Li N., Gu Y., Zhao D., Liao Q., Liu M. (2013). Mutations in GBA and risk of Parkinson’s disease: A meta-analysis based on 25 case control studies. Neurol. Res..

[81] Mallett V., Ross J.P., Alcalay R.N., Ambalavanan A., Sidransky E., Dion P.A., Rouleau G.A., Gan-Or Z. (2016). GBA P.T369M substitution in Parkinson disease: Polymorphism or association? A meta-analysis. Neurol. Genet..

[82] Scarciolla O., Brancati F., Valente E.M., Ferraris A., De Angelis M.V., Valbonesi S., Garavaglia B., Uncini A., Palka G., Stuppia L. (2007). Multiplex ligation-dependent probe amplification assay for simultaneous detection of Parkinson’s disease gene rearrangements. Mov. Disord..

[83] Darvish H., Movafagh A., Omrani M.D., Firouzabadi S.G., Azargashb E., Jamshidi J., Khaligh A., Haghnejad L., Naeini N.S., Talebi A. (2013). Detection of copy number changes in genes associated with Parkinson’s disease in Iranian patients. Neurosci. Lett..

[84] Keyser R.J., Lombard D., Veikondis R., Carr J., Bardien S. (2010). Analysis of exon dosage using MLPA in South African Parkinson’s disease patients. Neurogenetics.

[85] Santiago J.A., Bottero V., Potashkin J.A. (2018). Evaluation of RNA blood biomarkers in the Parkinson’s disease biomarkers program. Front. Aging Neurosci..

[86] Wang J., Hoekstra J.G., Zuo C., Cook T.J., Zhang J. (2013). Biomarkers of Parkinson’s disease: Current status and future perspectives. Drug Discov. Today.

[87] Karlsson M.K., Sharma P., Aasly J., Toft M., Skogar Ö., Sæbø S., Lönneborg A. (2013). Found in transcription: Accurate Parkinson’s disease classification in peripheral blood. J. Parkinsons. Dis..

[88] Starhof C., Hejl A.M., Heegaard N.H.H., Carlsen A.L., Burton M., Lilje B., Winge K. (2019). The biomarker potential of cell-free microRNA from cerebrospinal fluid in Parkinsonian Syndromes. Mov. Disord..

[89] Alieva A.K., Filatova E.V., Karabanov A.V., Illarioshkin S.N., Slominsky P.A., Shadrina M.I. (2015). Potential biomarkers of the earliest clinical stages of Parkinson’s disease. Parkinsons. Dis..

[90] El-Agnaf O.M.A., Salem S.A., Paleologou K.E., Curran M.D., Gibson M.J., Court J.A., Schlossmacher M.G., Allsop D. (2006). Detection of oligomeric forms of α-synuclein protein in human plasma as a potential biomarker for Parkinson’s disease. FASEB J..

[91] Constantinescu R., Mondello S. (2013). Cerebrospinal fluid biomarker candidates for Parkinsonian disorders. Front. Neurol..

[92] Foulds P.G., Mitchell J.D., Parker A., Turner R., Green G., Diggle P., Hasegawa M., Taylor M., Mann D., Allsop D. (2011). Phosphorylated α-synuclein can be detected in blood plasma and is potentially a useful biomarker for Parkinson’s disease. FASEB J..

[93] Wang Y., Shi M., Chung K.A., Zabetian C.P., Leverenz J.B., Berg D., Srulijes K., Trojanowski J.Q., Lee V.M.Y., Siderowf A.D. (2012). Phosphorylated α-synuclein in Parkinson’s disease. Sci. Transl. Med..

[94] Kang J.H., Irwin D.J., Chen-Plotkin A.S., Siderowf A., Caspell C., Coffey C.S., Waligórska T., Taylor P., Pan S., Frasier M. (2013). Association of cerebrospinal fluid β-amyloid 1-42, t-tau, p-tau 181, and α-synuclein levels with clinical features of drug-naive patients with early parkinson disease. JAMA Neurol..

[95] Younes-Mhenni S., Frih-Ayed M., Kerkeni A., Bost M., Chazot G. (2007). Peripheral Blood Markers of Oxidative Stress in Parkinson’s Disease. Eur. Neurol..

[96] Shi M., Bradner J., Hancock A.M., Chung K.A., Quinn J.F., Peskind E.R., Galasko D., Jankovic J., Zabetian C.P., Kim H.M. (2011). Cerebrospinal fluid biomarkers for Parkinson disease diagnosis and progression. Ann. Neurol..

[97] Shi M., Zabetian C.P., Hancock A.M., Ginghina C., Hong Z., Yearout D., Chung K.A., Quinn J.F., Peskind E.R., Galasko D. (2010). Significance and confounders of peripheral DJ-1 and alpha-synuclein in Parkinson’s disease. Neurosci. Lett..

[98] Lin X., Cook T.J., Zabetian C.P., Leverenz J.B., Peskind E.R., Hu S.C., Cain K.C., Pan C., Edgar J.S., Goodlett D.R. (2012). DJ-1 isoforms in whole blood as potential biomarkers of Parkinson disease. Sci. Rep..

[99] Han M., Nagele E., DeMarshall C., Acharya N., Nagele R. (2012). Diagnosis of parkinson’s disease based on disease-specific autoantibody profiles in human sera. PLoS ONE.

[100] Wu Y., Yao Q., Jiang G.-X., Wang G., Cheng Q. (2019). Identification of distinct blood-based biomarkers in early stage of Parkinson’s disease. Neurol. Sci..

[101] Pellecchia M.T., Santangelo G., Picillo M., Pivonello R., Longo K., Pivonello C., Vitale C., Amboni M., De Rosa A., Moccia M. (2014). Insulin-like growth factor-1 predicts cognitive functions at 2-year follow-up in early, drug-naïve Parkinson’s disease. Eur. J. Neurol..

[102] Swanson C.R., Berlyand Y., Xie S.X., Alcalay R.N., Chahine L.M., Chen-Plotkin A.S. (2015). Plasma apolipoprotein A1 associates with age at onset and motor severity in early Parkinson’s disease patients. Mov. Disord..

[103] Trifonova O., Lokhov P., Archakov A. (2013). Postgenomics diagnostics: Metabolomics approaches to human blood profiling. OMICS.

[104] Abe T., Isobe C., Murata T., Sato C., Tohgi H. (2003). Alteration of 8-hydroxyguanosine concentrations in the cerebrospinal fluid and serum from patients with Parkinson’s disease. Neurosci. Lett..

[105] Ascherio A., LeWitt P.A., Xu K., Eberly S., Watts A., Matson W.R., Marras C., Kieburtz K., Rudolph A., Bogdanov M.B. (2009). Urate as a predictor of the rate of clinical decline in Parkinson disease. Arch. Neurol..

[106] Goldstein D.S., Holmes C., Sharabi Y. (2012). Cerebrospinal fluid biomarkers of central catecholamine deficiency in Parkinson’s disease and other synucleinopathies. Brain.

[107] D’Andrea G., Pizzolato G., Gucciardi A., Stocchero M., Giordano G., Baraldi E., Leon A. (2019). Different Circulating Trace Amine Profiles in De Novo and Treated Parkinson’s Disease Patients. Sci. Rep..

[108] Bogdanov M., Matson W.R., Wang L., Matson T., Saunders-Pullman R., Bressman S.S., Beal M.F. (2008). Metabolomic profiling to develop blood biomarkers for Parkinson’s disease. Brain.

[109] Havelund J.F., Heegaard N.H.H., Færgeman N.J.K., Gramsbergen J.B. (2017). Biomarker research in parkinson’s disease using metabolite profiling. Metabolites.

[110] Koulman A., Tapper B.A., Fraser K., Cao M., Lane G.A., Rasmussen S. (2007). High-throughput direct-infusion ion trap mass spectrometry: A new method for metabolomics. Rapid Commun. Mass Spectrom..

[111] Lin L., Yu Q., Yan X., Hang W., Zheng J., Xing J., Huang B. (2010). Direct infusion mass spectrometry or liquid chromatography mass spectrometry for human metabonomics? A serum metabonomic study of kidney cancer. Analyst.

[112] Lokhov P.G., Dashtiev M.I. (2010). Metabolite profiling of blood plasma of patients with prostate cancer. Metabolomics.

[113] Lokhov P.G., Kharybin O.N., Archakov A.I. (2012). Diagnosis of lung cancer based on direct-infusion electrospray mass spectrometry of blood plasma metabolites. Int. J. Mass Spectrom..

[114] Lokhov P.G., Trifonova O.P., Maslov D.L., Balashova E.E., Archakov A.I., Shestakova E.A., Shestakova M.V., Dedov I.I. (2014). Diagnosing impaired glucose tolerance using direct infusion mass spectrometry of blood plasma. PLoS ONE.

[115] Lokhov P.G., Balashova E.E., Voskresenskaya A.A., Trifonova O.P., Maslov D.L., Archakov A.I. (2016). Mass spectrometric signatures of the blood plasma metabolome for disease diagnostics. Biomed. Rep..

[116] Lokhov P.G., Trifonova O.P., Maslov D.L., Archakov A.I. (2013). Blood plasma metabolites and the risk of developing lung cancer in Russia. Eur. J. Cancer Prev..

[117] Lokhov P.G., Balashova E.E., Trifonova O.P., Maslov D.L., Ponomarenko E.A., Archakov A.I. (2020). Mass spectrometry-based metabolomics analysis of obese patients’ blood plasma. Int. J. Mol. Sci..

[118] Balashova E.E., Lokhov P.G., Maslov D.L., Trifonova O.P., Khasanova D.M., Zalyalova Z.A., Nigmatullina R.R., Archakov A.I., Ugrumov M.V. (2017). Plasma Metabolome Signature in Patients with Early-stage Parkinson Disease. Curr. Metab..

[119] Rinne J.O., Anichtchik O.V., Eriksson K.S., Kaslin J., Tuomisto L., Kalimo H., Röyttä M., Panula P. (2002). Increased brain histamine levels in Parkinson’s disease but not in multiple system atrophy. J. Neurochem..

[120] Maslov D.L., Balashova E.E., Lokhov P.G., Archakov A.I. (2017). Pharmacometabonomics—The novel way to personalized drug therapy. Biomeditsinskaya Khimiya.

[121] Lokhov P.G., Maslov D.L., Balashova E.E., Trifonova O.P., Medvedeva N.V., Torkhovskaya T.I., Ipatova O.M., Archakov A.I., Malyshev P.P., Kukharchuk V.V. (2015). Mass spectrometry analysis of blood plasma lipidome as the method of disease diagnostics, evalution of effectiveness and optimization of drug therapy. Biochem. Suppl. Ser. B Biomed. Chem..

[122] Balashova E.E., Maslov D.L., Lokhov P.G. (2018). A metabolomics approach to pharmacotherapy personalization. J. Pers. Med..

[123] Trifonova O., Knight R.A., Lisitsa A., Melino G., Antonov A.V. (2016). Exploration of individuality in drug metabolism by high-throughput metabolomics: The fast line for personalized medicine. Drug Discov. Today.

[124] Furlanut M., Furlanut M., Benetello P. (2001). Monitoring of L-DOPA concentrations in Parkinson’s disease. Pharmacol. Res..

